# The B-VITAGE trial: A randomized trial of homocysteine lowering treatment of depression in later life

**DOI:** 10.1186/1745-6215-11-8

**Published:** 2010-01-25

**Authors:** Andrew H Ford, Leon Flicker, Kieran McCaul, Frank van Bockxmeer, Sarah Hegarty, Varsha Hirani, Stephen Fenner, Osvaldo P Almeida

**Affiliations:** 1 Western Australian Centre for Health & Ageing, Centre for Medical Research, University of Western Australia, Perth, Australia; 2 School of Psychiatry & Clinical Neurosciences, University of Western Australia, Perth, Australia; 3 Department of Psychiatry, Royal Perth Hospital, Perth, Australia; 4 School of Medicine & Pharmacology, University of Western Australia, Perth, Australia; 5 Department of Geriatric Medicine, Royal Perth Hospital, Perth, Australia; 6 School of Pathology & Laboratory Medicine, University of Western Australia, Perth, Australia; 7 Department of Biochemistry, Royal Perth Hospital, Perth, Australia

## Abstract

**Background:**

Depression is a leading cause of disability worldwide and depressive symptoms are common in later life. Observational evidence suggests that depression is more prevalent among people with high plasma homocysteine (tHcy), but the results of randomized trials to date have been unable to show that lowering tHcy through the supplementation of vitamins B_6_, B_12 _and folate benefits depressive symptoms. We designed the B-VITAGE trial to determine whether adjunctive treatment with vitamins B_6_, B_12 _and folate increases the efficacy of standard antidepressant treatment.

**Methods/Design:**

The B-VITAGE trial is a 12-month randomized, double-blind, placebo-controlled trial of daily citalopram (20 to 40 mg) plus B_12_(0.4 mg), B_6 _(25 mg) and folic acid (2 mg) or citalopram (20 to 40 mg) plus placebo for the treatment of depression in later life. The trial aims to recruit over 300 older adults with major depression (DSM-IV) and has been powered to detect the impact of an intervention associated with moderate effect size. Depressive symptoms will be rated with the Montgomery-Åsberg Depression Rating Scale (MADRS). The trial has two main outcomes of interest: a reduction of 50% or more in the MADRS total score between baseline and week 12 and the remission of the depressive episode at weeks 12, 26 and 52 according to DSM-IV criteria. We hypothesize that subjects randomly allocated to the vitamin arm of the study will be more likely to show a clinically significant improvement and achieve and maintain remission of symptoms at 12, 26 and 52 weeks. Secondary outcomes of interest include compliance with treatment, reduction in the severity of depressive symptoms, switching to different antidepressants, the use of non-pharmacological antidepressant treatments, response to treatment according to MTHFRC677T genotype, and changes in cognitive function over 52 weeks.

**Conclusions:**

The results of this trial will clarify whether the systematic use of B-vitamins improves the response of older adults to standard antidepressant treatment. We anticipate that our findings will have implications for clinical practice and health policy development.

**Trial Registration:**

The trial is registered with the Australian Clinical Trials Registry, trial number (())ACTRN12609000256279.

## Background

Depression is a leading cause of disability worldwide [1] and depressive symptoms are a common occurrence in later life [2]. The causes of depression in later life are likely to be varied and complex but current available evidence suggests that cardiovascular risk factors and diseases may be involved [3]. Total plasma homocysteine (tHcy) has been implicated as a risk factor for cardiovascular disease [4] and more recently associated with depression in later life [5].

The observation that B-vitamins and tHcy may be associated with depression was initially described in the 1970's by Reynolds and colleagues [6]. Substantial observational data from various sources has since been gathered in support of this association, including nine cross-sectional studies showing that older men and women with depression have higher tHcy than their non-depressed peers [5,7-14]. A recent meta-analysis of all observational studies [5] designed to investigate the association between tHcy and depression in later life showed that high plasma tHcy was associated with an increase in the odds of depression (OR = 1.70, 95%CI = 1.38 - 2.08; fixed effects model).

Homocysteine is a sulfur-containing amino acid derived from the essential amino acid methionine through demethylation [15]. Total plasma homocysteine can be reliably reduced by the simple supplementation of vitamins B_12_, B_6 _and folic acid [16] which are all involved in the metabolism of homocysteine through two different pathways.

Homocysteine can be remethylated to methionine via the 5-methyl-tetrahydrofolate pathway that is mediated by the 5,10-methylenetetrahydrofolate reductase (*MTHFR) *enzyme and requires B_12 _and folic acid, or condensed with serine to form cysthionine in a reaction that uses vitamin B_6 _[17]. Methionine is the immediate precursor of S-adenosylmethionine (SAM), the methyl donor of numerous methylation reactions in the brain, many of which are directly involved in the synthesis and metabolism of monoamines such as dopamine, norepinephrine and serotonin [9]. This suggests that the association between elevated homocysteine and depression is biologically plausible.

Another way of investigating the association between tHcy and depression is via the *MTHFR *gene. *MTHFR *converts 5,10-methylene tetrahydrofolate to 5-methyltetrahydrofolate which is needed for the remethylation of homocysteine to methionine. Approximately 10% of the population is homozygous for the 677 C→T polymorphism of the MTHFR gene. This polymorphism reduces the production of 5-methyltetrahydrofolate and thus increases total plasma homocysteine by around 20% (approximately 2 μmol/L) [18]. We examined the association between *MTHFR *C677T genotype, tHcy and clinical history of ever depression in the Health in Men Study (HIMS) cohort [5]. We found that tHcy was 1.4 μmol/L higher amongst TT than CC carriers. We then conducted a meta-analysis of studies investigating the association between depression and *MTHFR *genotype, including our own data from HIMS. Overall TT carriers had a 22% increase in the odds of depression compared with CC carriers (OR = 1.22, 95%CI = 1.01 - 1.47).

Observational evidence would therefore support a causal link between high plasma homocysteine and depression. The most compelling way to establish causality however, is by means of randomized controlled trials where depressed older adults are exposed to homocysteine-lowering treatment or placebo. Seven clinical trials reported the results of homocysteine-lowering treatment on the mood of people with depression [19-25]. Together, these studies evaluated the effect of some form of B-vitamin supplementation in a heterogeneous group of 788 patients, some of them without clinically significant depression. Their results suggest that folate supplementation may have a role to play in the adjunctive treatment of depression, although the number of patients studied to date is small and the reported benefits associated with B-vitamin treatment are mostly based on *post hoc *comparisons of subgroups of patients (for example, women but not men).

The results from clinical trials to date are therefore modest at best and do not adequately support the findings of observational research. These seven studies were small in size and some were obviously underpowered to detect smaller clinically significant changes in mood. The trials were heterogeneous in nature and recruited participants with a wide age range and severity of depressive symptoms. Older adults have a higher risk of cerebrovascular disease and are therefore a group that may benefit more from homocysteine-lowering treatment given the hypothesized link between cerebrovascular disease and depression [3]. Trial durations also varied and most available studies were too brief to produce useful information about the clinical implications of longer periods of supplementation.

The B-VITAGE trial aims to recruit a community-representative sample of over 300 older adults (age 60 and older) who will be randomly allocated to treatment with citalopram and vitamins or citalopram and placebo in a double blind fashion for 12 weeks. Citalopram was chosen due to its relatively low incidence of adverse effects, favorable safety profile and relatively low risk of drug interactions [26]. After 12 weeks, the antidepressant management of participants will be devolved to their general practitioner. Blinded treatment with vitamins and placebo will remain unchanged for 52 weeks.

We hypothesize that older adults randomized to receive citalopram and vitamins B_12_, B_6 _and folic acid will be more likely to show a clinically significant reduction in the severity of depressive symptoms over 12 weeks. Additionally, we hypothesize that participants in the vitamin group will be more likely to achieve and sustain remission of their depression over 12 months. The study will also aim to investigate whether *MTHFR *carrier status has any impact on response to supplementation with B vitamins (secondary aim).

## Methods/Design

### Study Design

The B-VITAGE trial is a 12-month randomized, double-blind, placebo-controlled trial of citalopram plus vitamin B_12_, B_6 _and folic acid or citalopram plus placebo for the treatment of depression in later life. The Human Research Ethics Committee of the Royal Perth Hospital approved the study protocol. Recruitment is currently ongoing. The trial is investigator driven and will be coordinated by the principle investigator (OPA). A steering committee consisting of the lead investigators, study coordinator and research assistant will meet on a monthly basis to oversee the running of the trial.

### Setting

Participants will be treated as outpatients at the Royal Perth Hospital Mood Disorder Clinic in Western Australia, Australia. Taxi vouchers will be provided for travel to and from the hospital if required. Remuneration for participants' time will not be provided.

### Eligibility Criteria

Participants will be included if they:

1. are aged 60 years or older,

2. satisfy the DSM-IV [27] criteria for a major depressive episode in the context of a major depressive disorder (recurrent or single episode),

3. have a Montgomery-Åsberg Depression Rating Scale (MADRS) [28] score of 20 or greater (moderate or severe depression),

4. are fluent in written and spoken English.

We will exclude from the trial older adults who:

1. show evidence of risky alcohol use as judged by an Alcohol Use Disorders Identification Test (AUDIT) [29] score greater than 15,

2. have Parkinson's disease or a documented clinical history of stroke,

3. show evidence of clinically significant cognitive impairment (Mini-Mental State Examination - MMSE [30] score lower than 24),

4. are actively suicidal or display prominent psychotic symptoms,

5. have a life-threatening illness that is likely to compromise 12-month survival,

6. are unable to follow the research schedule,

7. are currently being treated with a monoamine oxidase inhibitor or electroconvulsive therapy,

8. have a clinical history of an allergic reaction to citalopram or escitalopram,

9. do not provide written informed consent.

Participants taking an antidepressant will not be excluded provided they fulfil the entry criteria (i.e., fail to respond to a trial with another antidepressant) and agree to change the medication to citalopram. In this case, citalopram will be commenced after a one-week washout period to minimize carryover effects from the existing antidepressant while minimizing the risk of deterioration in the mental state of participants that could potentially occur with longer washout periods. We will also include in the study older adults taking vitamins as long as they agree to discontinue their use for the duration of the trial.

The local Ethics Committee questioned the inclusion of participants with a baseline serum B_12 _<140 pmol/L who drink alcohol, as they could potentially be randomly allocated to treatment with placebo. As a result of these discussions, the B-VITAGE trial will also exclude from participation older adults who have an AUDIT score greater than 7 and a serum concentration of methylmalonic acid (MMA) >0.4 μmol/L. MMA is a tissue marker of vitamin B12 deficiency [31].

### Screening and Recruitment Strategies

We have been using five different approaches to screen and recruit community-dwelling older adults with depression:

1. General practitioners in the Perth Metropolitan Area are currently being approached and asked to participate. They will be requested to send an invitation to all their older patients (60+) who have consulted them in the past 12 months. The mail out will include an invitation letter, the Patient Health Questionnaire (PHQ-9), [32] the AUDIT, a consent form and information about the study. Potential participants will be asked to return the completed PHQ-9, AUDIT and consent form to the study team if they wish to participate.

2. The Western Australian Centre for Health and Ageing have access to large databases of potential participants from previous studies (DEPS-GP, [33] n = 4305 and HIMS, [34] n ≈ 7500) and these will be utilized to aid recruitment. Participants from these studies who have indicated that they would be happy to have future contact with the Centre will be invited to participate.

3. Older Adult Mental Health Services in metropolitan Perth receive approximately 2000 referrals per year, of which roughly 1000 are due to a depressive episode. We estimate that approximately 500 of these patients will be eligible (estimate based on clinical audit of the Inner City Older Adult Mental Health Service) and that 30% will consent to participate. Referrals will therefore be sought from these services.

4. Public advertising campaign. The study will be widely publicized in the media including community newspapers. We will also be distributing flyers in some GP practices and community organizations.

5. The last recruitment strategy will involve contacting individual GPs and seeking referrals directly to the clinic. This will be offered as an assessment service and suitable consenting patients will be enrolled.

Anyone who screens positive on the PHQ (i.e. scores 10 or higher) will be contacted by the research staff upon receiving the questionnaire and invited to attend a clinic appointment within 2 weeks. Patients who decline to participate will be referred to their general practitioners who will be informed of the results of their screening questionnaires. We anticipate that the recruitment of participants will be completed by the end of 2011, and that the final endpoint of the study will be the end of 2012. Clinical records will be managed as per existing hospital policies and participant information kept confidential at all times.

### Objectives

#### 1) Clinically significant improvement in the severity of depressive symptoms

One primary outcome of interest in this study is a reduction of 50% or more in the MADRS total score between baseline and week 12 which is considered to be of clinical significance. Our hypothesis is that participants randomly allocated to treatment with vitamins will be more likely than placebo control subjects to show a clinically significant reduction in the severity of depressive symptoms between baseline and week 12.

#### 2) Remission from index depressive episode

The other primary outcome of interest in this trial is the remission of the depressive episode at weeks 12, 26 and 52 according to DSM-IV criteria i.e. the participant no longer meets the diagnostic criteria for a diagnosis of major depression. Our hypothesis is that participants in the vitamin group will be more likely to achieve and maintain remission of their depression.

Secondary outcomes of interest include compliance with treatment, changes in the MADRS scores from baseline to week 12 (i.e. difference between the groups in their mean change from baseline MADRS scores as a continuous measure), switching to different antidepressants, the use of non-pharmacological antidepressant treatment (such as psychotherapy), response to treatment according to MTHFRC677T genotype, and changes in cognitive function over 52 weeks.

### Outcomes

#### Determining eligibility

##### Depression

Potential participants will be screened with the PHQ-9, a widely used and well-accepted screening instrument for depression. A PHQ-9 total score of 10 or more is associated with 88% sensitivity and specificity for a diagnosis of a depressive episode according to DSM-IV criteria. The PHQ-9 is a simple and easy to use instrument that takes no longer than 3 minutes to complete.

##### Risk alcohol use

the AUDIT is a 10-item questionnaire designed to screen for excessive drinking. The scale takes 2 minutes to complete, with scores greater than 15 being indicative of alcohol dependency.

##### Cognitive impairment and function

we will use the Mini-Mental State Examination (MMSE) to assess the cognitive abilities of participants and this will be administered by the research staff during the baseline clinic assessment. Those with a score lower than 24 will be excluded from further participation due to the presence of cognitive impairment. In addition, trained research staff will administer the Consortium to Establish a Registry for Alzheimer's Disease (CERAD) [35] neuropsychology battery.

#### Establishing the diagnosis of a major depressive episode

We will use the Mini-International Neuropsychiatric Interview (M.I.N.I.) to assess the current mental state of participants as well as their prior mental health history [36]. The M.I.N.I. is a short structured clinical interview that can be administered by trained research staff. It provides all relevant information to establish the diagnosis of a depressive episode according to DSM-IV criteria.

#### Measuring the severity of depressive symptoms

Participants will undergo a psychiatric assessment with one of the three study psychiatrists who will use the Montgomery-Åsberg Depression Rating Scale (MADRS) to measure the severity of depressive symptoms. The MADRS is widely used in clinical trials of depression because it is demonstrably sensitive to change. The MADRS will be rated at baseline, week 4, 8, 12, 26 and 52. The scale takes approximately 10-15 minutes to rate.

The M.I.N.I. and MADRS will measure the two primary outcomes of interest (i.e. clinically relevant improvement and remission of depressive symptoms).

#### Medical morbidities, lifestyle and medications

We will collect information from participants regarding current and past medical history and current medications. We will also record basic demographic information (date of birth, marital status, educational achievement and living arrangements) and will measure weight, height, blood pressure and pulse rate using standard procedures.

#### Monitoring of adverse reactions and events

We will use the Systematic Assessment for Treatment Emergent Effects [37] (Saftee) to monitor for any adverse reactions associated with the use of citalopram and vitamins or placebo. For the purposes of this study this will involve a checklist of symptoms commonly associated with experimental medications and antidepressants in particular, such as tremor, headaches, anxiety, insomnia, gastrointestinal symptoms and sexual side-effects, as well questions about painful peripheral neuropathy which can rarely be associated with excessive vitamin B_6 _use [38]. The participant will be asked about the emergence of any new symptoms since the last assessment and these will be rated on a 4 point scale - not at all, a little, quite a bit and extremely. The checklist will be completed during each clinic visit. The investigators will use this to screen for any new symptoms which could be attributed to the study medication as well as to monitor for any changes in the pattern and intensity of symptoms.

An unblinded geriatrician (LF) will independently monitor the blood results and any significant adverse events. He will notify the participant and their general practitioner in the event of any concerns. In addition he will notify the Ethics Committee in the event of any serious adverse event and unblinding of the investigators will occur.

#### Biochemistry tests

Fasting blood samples will be collected at baseline and again at weeks 12, 26 and 52 for the assessment of serum B_12 _and red cell folate (measured by standard competitive assays using the Abbott Axsym analyzer), as well as plasma homocysteine (measured by reverse phase high performance liquid chromatography after treatment with tributylphosphine, deproteinization and fluorogenic derivatization using the method of Araki and Sako) [39].

#### Genetics

Genomic DNA will be isolated from nucleated blood cells via a Triton X-100 method, and the 677 C → T mutation will be determined by use of the polymerase chain reaction (PCR) and *Hinf*I restriction enzyme digestion as described by Frosst et al. [40]*Hinf*I digestion (1.5 U/25 μL reaction mixture) will be performed directly in the PCR tube at 37°C for 4 hours before analysis of restriction fragments by polyacrylamide gel electrophoresis (PAGE; 12% T, 3.3% C) [41]. Allele frequencies will be estimated by gene counting and observed numbers of each genotype will be compared with those expected under Hardy-Weinberg equilibrium. We will aim to clarify if the clinical response to vitamin treatment is affected by *MTHFR *genotype.

### Sample Size

We based our sample size calculations to test hypothesis 1 on the results of the trial published by Coppen and Bailey, [22] which is the largest study of adjunctive antidepressant treatment with folic acid published to date. This study also enrolled a community-representative sample although recruited adults 18 years and over. The B-VITAGE trial will therefore have the added potential advantage of having participants with higher average tHcy given their older age (60 plus). We have previously shown that tHcy increases with age and that response to homocysteine-lowering vitamins is higher in people with higher tHcy [17]. Fluoxetine and citalopram are both selective serotonin reuptake inhibitors (SSRI's) and have been shown to have similar efficacy in the treatment of major depression [42]. The addition of vitamins B_12 _and B_6 _in our study may provide additional benefit (and therefore potentially bigger effect sizes) than folate supplementation alone given their respective roles in homocysteine metabolism.

Coppen and Bailey reported that 64.7% of their subjects treated with fluoxetine 20 mg and folic acid 0.5 mg were free of clinically significant depressive symptoms at the end of 10 weeks (Hamilton Depression Rating Scale score = 9), compared with 48.3% of those receiving fluoxetine 20 mg and placebo. A difference in remission rates of this magnitude is considered to be of meaningful clinical significance [43]. A study with 310 subjects (155 per group) would have 80% power to demonstrate a difference of this magnitude, with 2-tailed alpha set at 5%. We conservatively estimate a 12-week loss to follow-up of 15%, with an additional 10% lost between weeks 12 and 52 (total loss of 25%). Therefore, we will aim to recruit 388 older adults with a depressive episode into the trial (194 subjects per group).

To estimate the power of our study to test hypothesis 2, we used previously published data showing that about 35% of older adults with depression either remain depressed or relapse during the first year of treatment [44]. A study with 155 subjects per group by the end of 12 months will have 81% power to declare as significant a between group difference of 15% (35% vs 20% for placebo and vitamins, respectively). Finally, a study of this size will have 80% power to declare as significant a mean difference of MADRS scores between the groups of 2.3 points (standard deviation = 7, alpha = 0.05) [45]. Changes in the scores of the MADRS between baseline and week 12 will constitute a secondary outcome of interest for the study.

### Randomization

Participants will be given consecutive numbers and will be allocated to treatment with citalopram plus B-vitamins or citalopram plus placebo according to a list of random numbers generated by computer in random permuted blocks of 6 to 16. This strategy decreases the risk of unblinding of participants in one particular block if, for some reason, the group membership of one of its members is disclosed. The list will be generated and maintained centrally by the Pharmacy at the Royal Perth Hospital until the last assessment of the study is completed.

### Intervention and Blinding

Eligible participants will be randomly allocated to treatment with citalopram plus 400 μg vitamin B_12_, 2 mg folic acid and 25 mg B_6 _or citalopram plus placebo. These doses have been shown to be effective in lowering homocysteine levels [17]. Citalopram will be introduced at a dose of 10 mg per day and increased to 20 mg per day after 2 weeks. After 4 weeks of treatment the dose of citalopram will be increased to 30 mg for those who still meet DSM-IV criteria for a depressive episode (i.e., partial or no response) and again to 40 mg at week 8, if appropriate (i.e., participant remains depressed and has not displayed significant adverse events that would rule out an increase in dose). After 12 weeks, the antidepressant management of participants will be devolved to their general practitioner. Blinded treatment with vitamins and placebo will remain unchanged for 52 weeks.

The Pharmacy will dispense commercially available citalopram tablets as prescribed by the study psychiatrists. Vitamins and placebo will be administered in the form of one daily oral capsule that will have the same shape, size, color, texture and taste. The capsules containing B-vitamins and placebo will be manufactured by Blackmores, Australia. The Pharmacy will be responsible for dispensing all study medication and will monitor compliance by pill counting. Compliance will be monitored further by regular assessment of the serum concentration of B12 and folate (these results will only be released to the blinded investigators by the Royal Perth Hospital Biochemistry Laboratory after the last study assessment is completed).

### Assessment Procedures

Participants will be asked to attend the study clinic on six occasions: baseline, 4, 8, 12, 26 and 52 weeks. Participants will be asked to complete a PHQ-9, AUDIT and consent form to screen for potential eligibility prior to attending a baseline assessment. Fasting blood will be collected at baseline as well as weeks 12, 26 and 52. A trained research assistant will administer an abbreviated (for the purpose of the study's objectives) version of the M.I.N.I. focussing on current and past episodes of depression, mania/hypomania, dysthymia and suicidality. All potential participants will then be assessed by one of the study psychiatrists who will administer the MADRS. The MADRS will be repeated during each visit whilst the M.I.N.I. will be administered at baseline, week 12, 26 and 52.

Participant's general practitioners will receive a letter from one of the study psychiatrists following each visit to keep them updated about the progress of their patient and to inform them of the outcome of the various assessments. Advice will be provided about further treatment if needed e.g. treatment of excessive alcohol use, alerting them to the presence of suicidal ideation or psychotic symptoms and suggesting further assessment of cognitive impairment. The table outlines the assessment schedule of the B-VITAGE trial (Table 1).

**Table 1:** Summary of assessments and procedures of the B-VITAGE Trial.

	Baseline	Week 4	Week 8	Week 12	Week 26	Week 52
PHQ-9	YES (screen)	--	--	--	--	--

AUDIT	YES (screen)	--	--	--	--	--

Morbidity	YES (screen)	--	--	--	--	--

Lifestyle & drugs	YES	--	--	YES	YES	YES

MMSE	YES	--	--	--	--	--

CERAD	YES			YES	YES	YES

MADRS	YES	YES	YES	YES	YES	YES

Saftee	YES	YES	YES	YES	YES	YES

Biochemistry	YES	--	--	YES	YES	YES

DNA	YES	--	--	--	--	--

Pill count	--	YES	YES	YES	YES	YES

Medication diary	--	YES	YES	YES	YES	YES

### Statistical Analysis

Analyses will be conducted based on completers and intention-to-treat. Hypotheses 1 and 2 will be tested using the Pearson's chi-square statistic (proportion of people in the active and placebo groups who show a reduction of 50% or more on the MADRS or who go into remission). To account for the potential effects of dropout, we will repeat our analysis using a mixed-effects model for repeated measures [46] to model the fixed categorical effects of treatment, time, and treatment by time interaction, as well as the continuous, fixed covariate of baseline MADRS score. In addition, missing data will be imputed via Imputation by Chained Equations (ICE) [47] in order to perform the intention-to-treat analysis. Data will be maintained, managed and analyzed using the statistical package Stata.

## Discussion

Depression is often a chronic and disabling condition [48] that does not always respond well to antidepressant treatment [49]. This trial will determine if the use of B-vitamins can improve the efficacy of antidepressant treatment amongst older adults with depression. Existing evidence provides some justification for the addition of folic acid to treatment with antidepressants [20,21,23] although these trials have generally been of short duration and have been powered to detect large effect sizes only. Previous studies have also enrolled younger participants who have not necessarily met criteria for major depression.

The design of this study has strengths and limitations that merit comment. Firstly, the study is not limited to older adults with elevated total plasma homocysteine and this may raise concerns about the power of the study. We have previously demonstrated that the use of B-vitamins reduces total plasma homocysteine amongst those with or without high homocysteine and those with or without low serum concentration of vitamins B_12 _and folate [17]. Consequently, the trial is unlikely to be limited by lack of efficacy of the intervention to reduce total plasma homocysteine. Secondly, it is possible that the 'depressogenic effect' of homocysteine is cumulative and that a 12-week trial is not sufficiently long to promote the changes associated with reduced tHcy (hypothesis 1). We cannot entirely dismiss this possibility, but currently available evidence from other trials of 6 to 10 weeks duration suggests this is unlikely to be the case [22-24]. In addition, the ongoing follow-up of participants for 12 months will enable us to monitor the longer term effects of B-vitamin supplementation on mood.

A third possible concern may be the impact of pre-existing antidepressant or vitamin treatment as well as other non-pharmacological treatments (e.g. psychotherapy) that participants may be exposed to. Whilst these may indeed have some impact on the outcomes for individual participants, the process of randomization should hopefully ensure an even distribution of these potential confounders in the two arms. Fourth, there is a possibility that discontinuation rates of vitamins may increase after the more intensive monitoring period of the first 12 weeks of the trial and rates may be higher in the intervention group presuming the hypothesis of improved outcomes in this group is accurate. This will be monitored via pill counts, blood tests and medication diaries with participants given prompts about compliance during each visit. Finally, questions may arise regarding the changes in antidepressant treatment that will take place after week 12 and how those may interfere with the outcomes of the trial. Changes in antidepressant treatment may not be randomly distributed between the two treatment groups and for this reason we will monitor changes of medications and dose for the duration of the trial.

These potential limitations should be seen in the context of the strengths of this trial. We will be enrolling older adults who are perhaps the group who may benefit most from homocysteine-lowering treatment. The trial has well-defined outcomes and is adequately powered to study these. The trial will be run over 12 months and will involve a community sample of participants recruited from a variety of clinical and non-clinical settings. All participants will meet the criteria for a major depressive episode.

It is envisaged that the results of this trial will add further evidence to the homocysteine-depression link and potentially help guide treatment decisions for older adults with depression.

## Competing interests

The authors declare that they have no competing interests.

## Authors' contributions

Study concept and design: OPA, KM and LF. Acquisition of data: OPA, AHF, SH, VH, SF, FvB, LF. Drafting of the manuscript: OPA and AHF. Critical revision of the manuscript for important intellectual content: AHF, LF, KM, FvB, SH, VH and OPA.
