# The B-VITAGE trial: A randomized trial of homocysteine lowering treatment of depression in later life

**DOI:** 10.1186/1745-6215-12-193

**Published:** 2011-08-16

**Authors:** Andrew H Ford, Leon Flicker, Kieran McCaul, Frank van Bockxmeer, Sarah Hegarty, Varsha Hirani, Stephen Fenner, Osvaldo P Almeida

**Affiliations:** 1 Western Australian Centre for Health & Ageing, Centre for Medical Research, University of Western Australia, Perth, Australia; 2 School of Psychiatry & Clinical Neurosciences, University of Western Australia, Perth, Australia; 3 Department of Psychiatry, Royal Perth Hospital, Perth, Australia; 4 School of Medicine & Pharmacology, University of Western Australia, Perth, Australia; 5 Department of Geriatric Medicine, Royal Perth Hospital, Perth, Australia; 6 School of Pathology & Laboratory Medicine, University of Western Australia, Perth, Australia; 7 Department of Biochemistry, Royal Perth Hospital, Perth, Australia

## Correction

Following the publication of our article [1], we noticed an error regarding the dosage of vitamin B12. In the manuscript, we reported that the daily dosage of vitamin B12 would be 0.4 mg, when in fact the dosage of vitamin B12 used in the B-VITAGE trial is 0.5 mg per day.

In the abstract:

The B-VITAGE trial is a 12-month randomized, double-blind, placebo-controlled trial of daily citalopram (20 to 40 mg) plus B_12_(0.4 mg), B_6 _(25 mg) and folic acid (2 mg) or citalopram (20 to 40 mg) plus placebo for the treatment of depression in later life.

Should read

The B-VITAGE trial is a 12-month randomized, double-blind, placebo-controlled trial of daily citalopram (20 to 40 mg) plus B_12_(0.5 mg), B_6 _(25 mg) and folic acid (2 mg) or citalopram (20 to 40 mg) plus placebo for the treatment of depression in later life.

In the "Intervention and Blinding" section:

Eligible participants will be randomly allocated to treatment with citalopram plus 400 μg vitamin B_12_, 2 mg folic acid and 25 mg B_6 _or citalopram plus placebo.

Should read

Eligible participants will be randomly allocated to treatment with citalopram plus 500 μg vitamin B_12_, 2 mg folic acid and 25 mg B_6 _or citalopram plus placebo.

## References

[B1] Ford AH Flicker L McCaul K van Bockxmeer F Hegarty S Hirani V Fenner S Almeida OP The B-VITAGE trial: A randomized trial of homocysteine lowering treatment of depression in later life Trials 2010 11 8 10.1186/1745-6215-11-8 20096138 PMC2822769

